# Occupational Hydrofluoric Acid Injury from Car and Truck Washing — Washington State, 2001–2013

**DOI:** 10.15585/mmwr.mm6432a4

**Published:** 2015-08-21

**Authors:** Carolyn K. Reeb-Whitaker, Carly M. Eckert, Naomi J. Anderson, David K. Bonauto

**Affiliations:** 1 Safety and Health Assessment and Research for Prevention Program, Washington State Department of Labor and Industries; 2 Department of Environmental and Occupational Health Sciences, University of Washington

Exposure to hydrofluoric acid (HF) causes corrosive chemical burns and potentially fatal systemic toxicity. Car and truck wash cleaning products, rust removers, and aluminum brighteners often contain HF because it is efficient in breaking down roadway matter. The death of a truck wash worker from ingestion of an HF-based wash product and 48 occupational HF burn cases associated with car and truck washing in Washington State during 2001–2013 are summarized in this report. Among seven hospitalized workers, two required surgery, and all but one worker returned to the job. Among 48 injured workers, job titles were primarily auto detailer, car wash worker, truck wash worker, and truck driver. Because HF exposure can result in potentially severe health outcomes, efforts to identify less hazardous alternatives to HF-based industrial wash products are warranted.

HF (Chemical Abstracts Service [CAS] no. 7664-39-3) can produce serious health effects through any exposure route. Exposure of HF solution to the eye can cause irritation as well as potentially permanent ocular damage. Tissue damage from skin contact occurs by two mechanisms. Free hydrogen ions can cause a corrosive burn, and free fluoride ions can cause local cellular destruction and penetrate the skin, causing muscle and bone necrosis. HF is insidiously toxic at the low concentrations (<20%) used in vehicle washing, because no overt corrosive skin burn is present at these concentrations and no initial pain alerts the worker to the exposure (1–3). Numbness, induced by the nerve damage resulting from fluoride ion penetration, leaves the injured worker unaware of the underlying necrosis that can progress for up to 24 hours after exposure (1,2). Systemically, fluoride toxicity by any route of exposure can cause fatal cardiac arrhythmias precipitated by hypocalcemia and hyperkalemia. Topical application and subcutaneous administration of calcium or magnesium compounds can be used to quench fluoride ions and preempt tissue damage.

Injuries in Washington State during 2001–2013 that met the case definition for exposure to HF among workers engaged in car or truck washing, including auto detailing, were identified through a number of sources. The single fatality was identified from Washington’s Division of Occupational Safety and Health (WA-DOSH) program. The seven hospitalized patients with burns were identified through Washington’s hospitalized occupational burn notifiable conditions rule. The 41 nonhospitalized workers with burns were identified through Washington’s State Fund workers’ compensation data system (4). Washington’s law mandates workers’ compensation insurance coverage for all employers, with 97.7% of employers and approximately two thirds of the state workforce insured through the Washington State Fund. Potential nonhospitalized burn patients were identified using the following Occupational Injury and Illness Classification System injury nature codes assigned to workers’ compensation claims: 050 (burns unspecified), 051 (chemical burns), 058 (multiple types of burns), and 059 (burns not elsewhere classified) (5). Among potential cases in both hospitalized and nonhospitalized workers, HF exposure (versus exposure to other or unspecified acids) during car or truck washing was confirmed through review of employer, worker, and/or physician narrative statements in the workers’ compensation medical record. Exposure information, including product Safety Data Sheets, were obtained from WA-DOSH inspection records or the medical record. Time-loss payments begin when work is missed on the fourth calendar day after the date of injury.

In 2012, a truck wash worker aged 38 years died after ingestion of a HF-based truck wash solution.* The victim placed a call to 911 emergency medical services; his 5-hour emergency department course was consistent with previous case reports of HF ingestion, including recurrent ventricular dysrhythmias (6). The product ingested was Fast Bright (NW Chemical, LLC) containing HF at <12% and sulfuric acid at <20% concentrations, with a pH of 1.5–1.6. The product is diluted before use on trucks, and the employer reported a dilution ratio resulting in a solution concentration of 0.65% HF. Both the concentrated and diluted solutions were present in the workplace, and it is not known which was ingested.

Workers’ compensation data from 2001–2013 were reviewed, and 48 HF chemical burn cases were identified. The median age of injured workers was 29 years (range = 15–62 years), three were female, and burn depth included superficial (first-degree), partial-thickness (second-degree), and full-thickness (third-degree) from exposure to products that ranged from 0.5% to 20% HF. HF concentration might have a greater effect on burn severity than the affected total body surface area burned. Eight workers (17%) received a median of 21 days (range = 2–40 days) in time-loss compensation.

Medical and contextual case details are summarized for the seven hospitalized workers (Table 1). Two required operative intervention, including burn debridement (case 1), split thickness skin graft (case 1), and escharotomy (case 3). Five injuries involved the fingers and hands. At the time of injury, workers wore improper gloves (e.g., cotton gloves) (case 2) or compromised gloves (with holes) (case 3). Two workers (cases 4 and 7) wore no gloves, one of whom manually washed a truck with an HF saturated washing mitt. One worker (case 6) had chemically resistant gloves and a face shield, but while scrubbing carwash walls overhead, the solution dripped down the brush handle and onto the worker’s arm and body. Delay in recognizing the exposure and in seeking medical attention occurred among nearly all hospitalized workers. Although immediate calcium gluconate administration can minimize the local and potential systemic effects of HF, no injured worker received calcium gluconate at their workplace. (Although the federal Occupational Safety and Health Administration (OSHA) and WA-DOSH require employers to provide a safe workplace, no regulation specifies that calcium gluconate be kept at the worksite.) With the exception of one worker (aged 15 years), all hospitalized workers returned to work; two (cases 1 and 7) received time-loss compensation, and two (cases 1 and 3) received permanent partial disability awards.

As a case example, one worker (case 1) splashed his left leg while transferring a cleaning solution of HF and sulfuric acid between containers. He did not irrigate the area and continued to work for approximately 1.5 hours with soaked pants and shoe until he developed an uncomfortable burning sensation. Upon evaluation, the patient was reported to have a quarter-sized brown necrotic area on the anterior left ankle and burn to the anterior left lower leg. Emergency medical technicians irrigated the area with calcium gluconate and transported him to a burn unit, where he received a calcium gluconate injection. He sustained a small area of full-thickness skin loss requiring excision and debridement with a skin graft. The worker received outpatient burn therapy and returned to part-time work 6 weeks after the injury. A foot paresthesia developed, and the worker received a permanent partial disability payment.

Body regions involved in the 41 nonhospitalized burn patients were upper extremity (16 patients, including hands and fingers [14]), head (14 patients, including eyes [14]), lower extremity (seven), multiple body regions (three), and trunk (one).

The exposed population includes workers in 16 industries (Table 2), with nearly half (n = 24) occurring in car washes (North American Industry Classification System [NAICS] no. 811192), which includes truck, van and trailer washing as well as auto detailing (7). HF burn injury also commonly occurred in new car dealers (NAICS no. 441110) (n = seven). Truck drivers (n = five) are at risk; three of the seven hospitalized cases were in truck drivers.

Workers apply HF-based solutions to vehicles with hand-held sprayers, pressurized metered sprayers, and open wash buckets. In addition to ready-to-use products, car and truck washes dilute concentrated HF-based products with water onsite to create the ‘use dilution’ solution, and exposure can occur during dilution and product transfer. Eight products were named in association with the 17 HF burn patients (Table 3). HF-based products often include additional chemicals that can burn, including sulfuric acid and phosphoric acid. Two products contained ammonium bifluoride (NH_4_HF_2_, CAS no. 1341-49-7), a chemical that dissociates into HF when dissolved in water and therefore has similar toxicity.

## Discussion

During 2001–2013, one fatal HF ingestion and 48 chemical burns from exposure to HF associated with car and truck washing were reported in Washington State. Although an estimated 134,000 workers are employed in the car wash industry (NAICS no. 811192) in the United States (8), few case reports of HF exposure in car and truck wash workers have been published. In a study that examined nine fatal unintentional occupational HF poisonings investigated by OSHA, none were found to be associated with car or truck washing (9). An Oregon-OSHA hazard alert† on HF exposure describes two car wash workers with HF burns, one of whom sustained a finger amputation (10). The broad distribution of HF burns associated with vehicle washing but occurring outside of the car wash industry suggests a large population of at-risk workers.

Less hazardous alternatives to HF-based wash products are available, and product substitution could have averted the HF burn injuries described in this report (3). When HF-based products are used, workplaces must use engineering and administrative controls to limit exposure. Product Safety Data Sheets reflect the hazardous nature of the product, and employers are faced with the challenge of managing exposure through worker training and use of personal protective equipment (PPE). However, appropriate PPE does not ensure protection; approximately nine of the cases described in this report involved failure of PPE, when product dripped inside rubber boots or gloves, permeated torn resistant gloves, or was sprayed up under safety glasses. Additionally, injury prevention efforts should include education and training with chemical manufacturers and distributors of HF-based products as well as the end users. Among the six identified products, one (made by Zep, Inc.) was produced internationally, and the rest were manufactured and distributed locally.

The findings in this report are subject to at least two limitations. First, groups exempted from Washington’s mandatory workers’ compensation law, including self-insured qualified employers, large employers, and sole proprietors, are not represented in the findings. Second, workers who have workers’ compensation coverage but do not file a claim would not be included. Barriers to accessing the workers’ compensation system include a lack of knowledge of the system, language other than English, beliefs about eligibility, and fear of job loss or retribution (10).

Occupational exposure to HF-based wash solutions can result in chemical burns, disability, and death. HF’s potential to cause severe injury combined with the inherent challenge of relying on PPE to protect workers warrants efforts to identify less hazardous alternatives, which would provide the most effective means of prevention.

**Summary**

What is already known on this topic?

Hydrofluoric acid (HF) causes chemical burns and is a serious systemic poison by all routes of exposure. HF is a chemical component in car and truck wash products, such as rust removers, aluminum brighteners, and wash formulations, because it is inexpensive and highly effective.

What is added by this report?

During 2001–2013, one death and 48 chemical burns from exposure to HF-based products used during car and truck washing, including auto detailing, were reported in Washington. The burns resulted in hospitalization, time lost from work, and disability. Reported diluted-use concentrations were <1% HF, and reported concentrated formulations contained up to 20% HF; both concentrations are hazardous to workers.

What are the implications for public health practice?

Because exposure to HF is toxic and can result in severe health outcomes, efforts to identify less hazardous alternatives to HF-based wash products are warranted. Further characterization of chemical burns from exposure to HF in auto detailers, car and truck wash workers, and truck drivers from other data sources or states would elucidate the magnitude and severity of this occupational health hazard.

## Figures and Tables

**TABLE 1:** Summary of cases of hydrofluoric acid exposure occurring during commercial car and truck washing — Washington, 2001–2013

Date of incident	Age*	Assigned task	Burn location	Burn classification (degree)†	Time loss (days)
Dec 2012	38	Wash truck	Systemic ingestion	—	Patient died
Feb 2001	23	Transfer solution	Left ankle, leg	3rd	40
Dec 2002	62	Wash trailer	Bilateral hands	2nd	0
Sep 2003	45	Wash truck	Right fingers (4 and 5)	3rd	0
Aug 2006	53	Wash wheels	Bilateral hands	Not reported	0
Jan 2007	15	Clean aluminum truck surfaces	Right thigh	3rd	0
May 2012	21	Wash walls and ceiling	Hands, legs, abdomen	1st	0
Mar 2013	32	Clean truck	Right thumb	2nd	16

* The fatality and all cases requiring hospitalization occurred in male workers.

† As reported by the physician in the medical record.

**TABLE 2:** Industry and job titles associated with all hydrofluoric acid burns — Washington, 2001–2013

NAICS no.	Industry description	Job title* (no. of workers affected)	No. of cases
811192	Car washes	Auto detailer (5), auto detail manager (1), car washer (5), car wash manager (4), truck washer (7), truck wash manager (1), washer (1)	24
441110	New car dealers	Auto detailer (6), dealership lot attendant (1)	7
238990	All other specialty trade contractors	Trucking manager, unknown	2
327320	Ready mix concrete manufacturing	Truck driver, mixer driver	2
561790	Other services to buildings and dwellings	Truck washer, cleaner	2
811310	Commercial and industrial machine and equipment (except auto and electronic) repair and maintenance	Mechanic, truck washer	2
111219	Other vegetable and melon farming	Unknown	1
113310	Logging	Truck driver	1
423830	Industrial machinery and equipment merchant wholesalers	Car washer	1
484121	General freight trucking, long distance, truckload	Mechanic	1
484210	Used household and office goods moving	Truck washer	1
484220	Specialized freight (except used goods) trucking, local	Truck driver	1
532111	Passenger car rental	Auto detailer	1
561320	Temporary help services	Mechanic	1
561431	Private mail centers	Truck driver	1
611512	Flight training	Truck washer	1
**Total no. of cases, including fatality**			**49**

**Abbreviation:** NAICS: North American Industry Classification System.

* Job title as given on the workers’ compensation Report of Accident form (free text).

**TABLE 3:** Car and truck wash products associated with 17 hydrofluoric acid (HF) burns — Washington, 2001–2013

Product	Manufacturer	No. of cases	HF% concentrate*	HF% dilute solution†
Zep-A-Lume	Zep, Inc.	6	5–10	4.2–8.3
Aluma Brite	—	3	—	—
Aluma-Kleen 1000	Wesmar Co., Inc.	2	10–20§	—
Fast Bright	NW Chemical, LLC	2	<12	0.65
A-Wall	CH_2_O, Inc.	1	—	0.5
Lume Brite Aluminum Cleaner and Brightener	—	1	<12	—
TC-303 Acid Aluminum Truck Brightener	Malco Products, Inc.	1	<5+ <4¶	—
Wheel Bright	Armor Chemical, Co.	1	—	7

* HF% concentrate is that reported on the product’s Safety Data Sheet.

† HF% dilute solution is self-reported by the worker or their employer in the medical record or during inspection by Washington’s Division of Occupational Safety and Health.

§ Product does not contain HF. It contains 10%–20% ammonium bifluoride (Chemical Abstracts Service no. 1341-49-7 [NH_4_HF_2_]), which dissociates into HF when dissolved in water.

¶ Product contains <5% HF and <4% ammonium bifluoride.

## Footnotes

* Whether this ingestion was intentional, inadvertent, or attempted self-harm is unknown.

† Available at http://www.orosha.org/pdf/hazards/2993-22.pdf.
